# Photochemical Synthesis and Properties of 1,6- and 1,8-Naphthalenophanes

**DOI:** 10.3390/molecules18011314

**Published:** 2013-01-21

**Authors:** Pablo Wessig, Annika Matthes

**Affiliations:** Institut für Chemie, Universität Potsdam, Karl-Liebknecht-Str. 24-25, D-14476 Potsdam, Germany

**Keywords:** photo-dehydro-Diels-Alder reaction, naphthalenophanes, atropisomerism, dynamic NMR, dynamic HPLC

## Abstract

Various 1,6- and 1,8-naphthalenophanes were synthesized by using the Photo-Dehydro-Diels-Alder (PDDA) reaction of bis-ynones. These compounds are easily accessible from ω-(3-iodophenyl)carboxylic acids in three steps. The obtained naphthalenophanes are axially chiral and the activation barrier for the atropisomerization could be determined in some cases by means of dynamic NMR (DNMR) and/or dynamic HPLC (DHPLC) experiments.

## 1. Introduction

**Figure 1:**
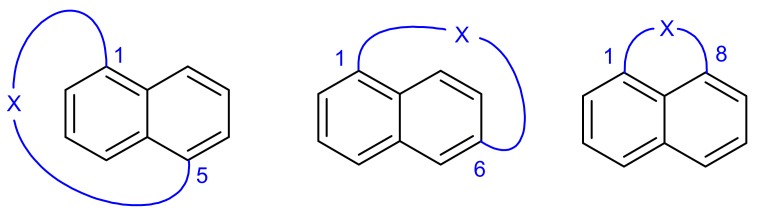
1,5-, 1,6- and 1,8-Naphthalenophanes.

Molecules consisting of an aromatic unit and a chain (which may contain further aromatic moieties) bridging two nonadjacent positions of the aromatic unit are referred to as cyclophanes [1,2]. Owing to their unusual molecular topology cyclophanes are often highly strained and, consequently, special methods are required for their synthesis. On the other site, many cyclophanes may be regarded as macrocyclic compounds and therefore often synthetic problems arise which are typical for macrocyclization reactions. Naphthalenophanes are a special class of cyclophanes where the central aromatic unit is a naphthalene moiety [3,4]. Recently we reported on a photochemical method for the preparation of axially chiral 1,5-naphthalenophanes and investigated their photophysical and chiroptical properties, as well as their ring strain [5]. This synthetic approach differs from most other routes to naphthalenophanes in that the naphthalene moiety is not present in the reactants but is formed in the cyclization step by means of the Photo-Dehydro-Diels-Alder (PDDA) reaction [6,7], which is a special case of the Dehydro-Diels-Alder (DDA) reaction [8]. In recent years we have intensively investigated the PDDA reaction and established it as a powerful method for the preparation of biaryls [9,10,11,12,13]. Herein we report in continuation of the above mentioned work on the synthesis of 1,6- and 1,8-naphthalenophanes (Figure 1).

## 2. Results and Discussion

### 2.1. Synthesis of PDDA Reactants

**Scheme 1:**
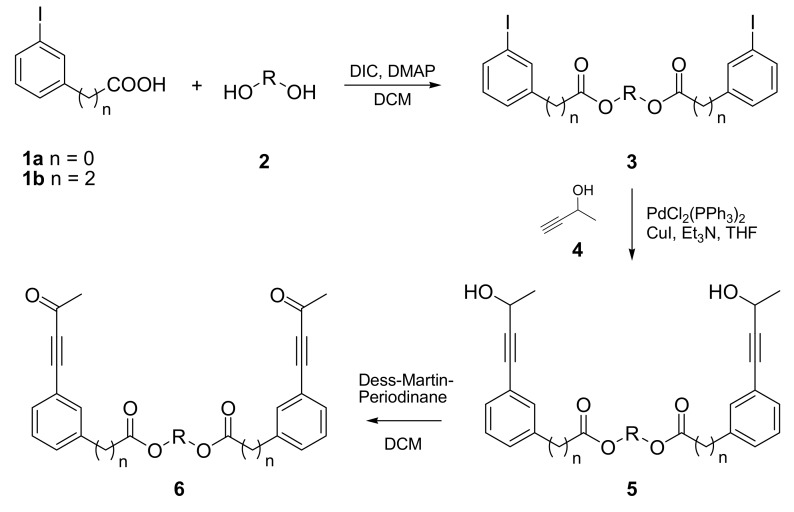
Synthesis of PDDA reactants **6**.

The synthesis of PDDA reactants was accomplished in three steps starting with commercially available 3-iodobenzoic acid (**1a**) and with 3-(3-iodophenyl)propionic acid (**1b**), respectively. The latter one was prepared from 3-iodobenzaldehyde and Meldrum’s acid according to Kawasaki *et al.* [14]. In the first step acids **1** were esterified with various diols **2** (for R see Table 1) using Steglich’s method [15]. The diesters **3** obtained in this way were subjected to a Sonogashira coupling [16] with 3-butyn-2-ol (**4**) under standard catalytic conditions giving compounds **5**. In the third step the secondary hydroxyl groups in **5** were oxidized with Dess-Martin periodinane [17,18] yielding bisynones **6** (Scheme 1). The yields of these three steps are almost always good to excellent and are summarized in Table 1.

### 2.2. Photochemical Synthesis of Naphthalenophanes

**Scheme 2:**
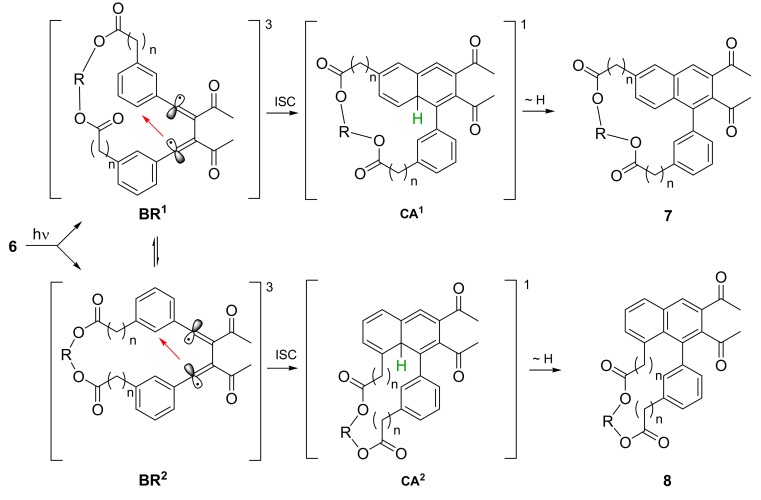
Photochemical formation of 1,6-naphthalenophanes **7** and 1,8-naphthalenophanes **8**.

It is well known that the PDDA reaction of bisynones proceeds, in contrast to the Diels-Alder reaction, not in a concerted way but in several discrete steps. After n-π* excitation an intersystem crossing (ISC) to the triplet state takes place, followed by the first C-C bond formation. The resulting triplet 1,3-butadien-1,4-diyl biradicals **BR** retain a certain conformational flexibility. Because the *ortho* positions with respect to the radical centers are not identical, two different approaches are possible in the second C-C bond forming step (Scheme 2, red arrows). 

**Scheme 3:**
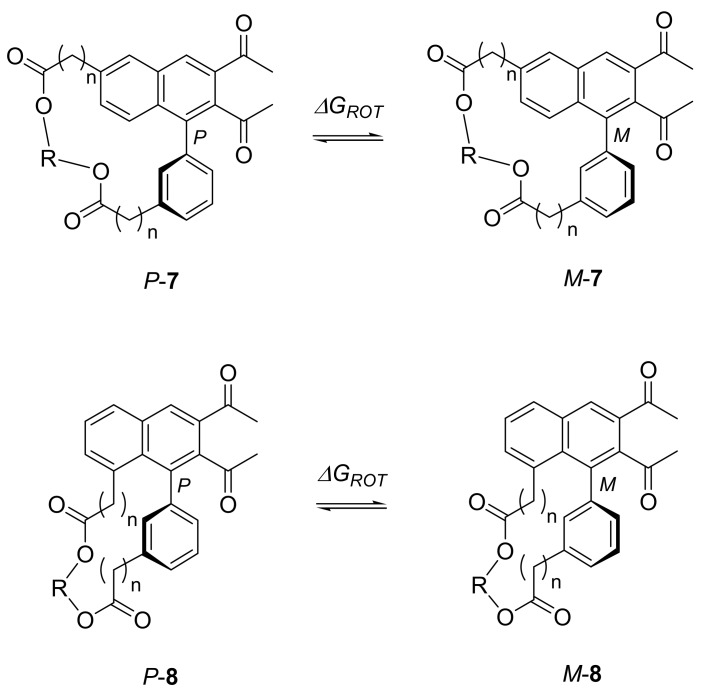
Atropisomerism of naphthalenophanes **7** and **8**.

Accordingly, the biradical conformers **BR^1^** react after ISC to cycloallenes **CA^1^**, whereas the conformers **BR^2^** give cycloallenes **CA^2^**. In the last step a formal 1,3-hydrogen migration takes place which is mediated by the solvent in most cases [11]. As a result, 1,6-naphthalenophanes **7** are formed from cycloallenes **CA^1^** and 1,8-naphthalenophanes **8** are the products of cycloallenes **CA^2^** (Scheme 3). The yields of naphthalenophanes are summarized in Table 2.

For comparative examination of naphthalenophanes, we defined k as the overall number of atoms forming the bridging chain (see Table 2). Only in the case of **6a** bearing the shortest chain (k = 9), we could not isolate a naphthalenophane from the complex product mixture, though we cannot rule out its formation. If the chain becomes longer, the products were obtained with partly remarkable yields. Except for **7e**/**8e** and **7f**/**8f** we were able to separate products **7** and **8** by flash chromatography. The respective structural assignment could be unambiguously performed by NMR spectroscopy. The regioselectivity was rather moderate despite in two cases (compounds **8b**,**c**) the formation of 1,8-naphthalenophanes is preferred. The low yield of 1,6-naphthalenophane **7b** could be explained by a considerable ring strain, whereas unfavorable repulsive H-H interactions of the alkyl chain should be responsible for the low overall yields of naphthalenophanes **7e**/**8e**. This phenomenon is well known for medium sized rings [19].

### 2.3. Atropisomerization of Naphthalenophanes ***7*** and ***8***

Naphthalenophanes **7** and **8** possess a chirality axis and may, therefore, form two different atropisomers *P* and *M* (Scheme 3). Axially chiral biaryl moieties are regularly found in natural products and are widely used as chiral catalysts and ligands in asymmetric synthesis. [20] For these applications it is of crucial importance to know the activation barrier of the atropisomerization *ΔG_ROT_*. Owing to the structural novelty of the naphthalenophanes described herein we were interested in the rotational barrier of these compounds. According to Bringmann [21] atropisomeric biaryls are configurationally stable at room temperature, if at least three bulky residues are bonded in the *ortho*-positions with respect to the chirality axis. In naphthalenophanes **7** and **8** only two of these residues exist and the question arose whether the atropisomers of these compounds are separable.

Several methods for the experimental determination of *ΔG_ROT_* are known, which complement one another. Which method is appropriate depends on the height of the barrier. If the barrier amounts to 15–20 kcal/mol, dynamic NMR (DNMR) [22] is the method of choice. The determination of considerably higher or lower barriers with this method is limited by the experimentally accessible temperature range with common NMR equipment and solvents. The DNMR method is based on the determination of the coalescence temperature of separated signals of enantiotopic hydrogens. If the barrier clearly exceeds 20 kcal/mol the dynamic HPLC (DHPLC) [23] comes into operation. Provided that a chiral compound is separated on a chiral stationary HPLC phase (which is not necessarily given) the method is based on the analysis of a plateau between the peaks of the individual enantiomers, which arise from partial racemization.

**Figure 2:**
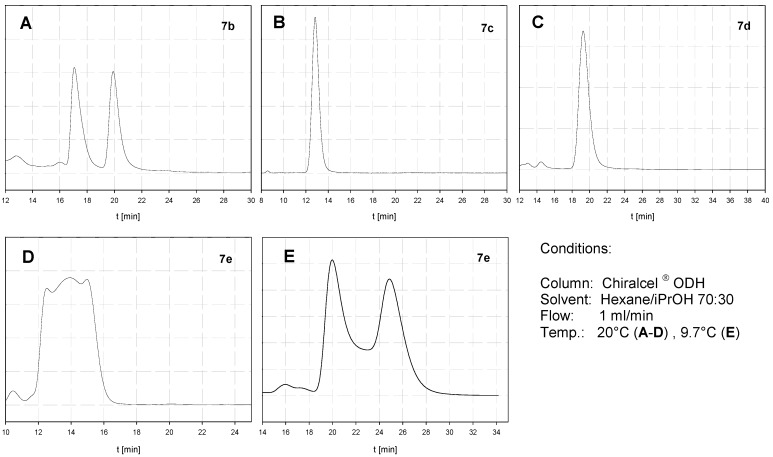
Chiral HPLC of 1,6-Naphthalenophanes **7b**–**e**.

Representative HPL chromatograms for 1,6-naphthalenophanes are depicted in Figure 2 demonstrating three different cases (a Chiralcel^®^ ODH column was used as chiral stationary phase). The enantiomers of compound **7b** are separated (**A**) and no racemization was observed even at elevated temperatures. Therefore **7b** may be regarded as configurationally stable at room temperature and the determination of rotational barrier cannot be performed neither by DHPLC nor DNMR. Compounds **7c** and **7d** (**B**,**C**) exhibit, even after excessive variation of the conditions, only one peak. There may be two reasons for this behavior: either the enantiomers of these compounds are not separated on this column or the rotational barrier is substantially lower than 20 kcal/mol (which is not very likely). In these cases we succeeded with DNMR experiments (*vide infra*). The HPL chromatogram of **7e** (**D**) exhibits at room temperature a broad peak with three maxima, which points to a partial racemization. Upon cooling to 9.7 °C the enantiomers are separated, though a plateau between the peaks is discernable (**E**). This enables the calculation of the rotational barrier to be 20.4 kcal/mol using the unified equation developed by Trapp [23]. The barriers for **8c**–**e** were determined in a similar manner. 

**Figure 3:**
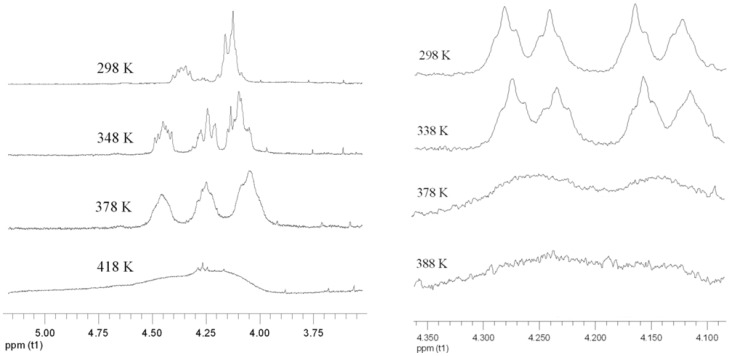
DNMR experiments of **7c** (left) and **7d** (right) in 1,1,2,2-tetrachloroethane-d^2^.

The determination of the rotational barrier using DNMR is exemplarily demonstrated for **7c** and **7d **(Figure 3). In both cases the signals of methylene groups adjacent to ester oxygen atoms are suitable for application of the method. For **7c** we observed a coalescence temperature of 418 K, whereas for **7d** the coalescence of the signals were reached at 398 K. Accordingly, the rotational barriers were calculated to be 20.6 kcal/mol (**7c**) and 19.6 kcal/mol (**7d**) respectively. The enantiomers of compound **8b** could not be separated by chiral HPLC and DNMR experiments failed due to insufficient quantities of this substance. Compound **8c** represents a special case. Even cooling of the column to 4 °C (the experimental lower limit) does not result in the formation of a plateau but only in broadening of the peaks. Therefore the barrier must be lower than 20 kcal/mol. On the other hand, DNMR experiments reach at 418 K the upper experimental limit without complete coalescence of the signals. Because the signals already considerably broadened, we estimate a coalescence temperature of 430 K giving a rotational barrier of about 20 kcal/mol. The determined barriers are summarized in Table 3. The slightly increasing barrier from **8c** to **8e** could be explained by steric hindrance of medium-sized rings.

## 3. Experimental

### 3.1. General

All products were purified by flash chromatography using silica gel 60 (230–400 mesh). For the analytical thin layer chromatography TLC aluminium sheets (silica gel 60 F_254_) were used. ^1^H-NMR spectra were recorded at 300 MHz and ^13^C-NMR spectra at 75 MHz in CDCl_3_ with a Bruker Avance 300 MHz Spectrometer. DNMR experiments were carried out in 1,1,2,2-tetrachloroethane-d^2^. Mass spectra were recorded with ESI-Q-TOF micro (quadrupole time-of-flight). Enantiomers were separated with Chiracel OD-H column using a Knauer HPLC Pump 64 and the detection occurred with Waters 996 Photodiode Array Detector. For solvents and temperatures see Figure 2.

### 3.2. General Procedure—Irradiation of Compounds ***6***

The diketones **6** were dissolved in *t*-butanol (350 mL) and acetone (10 mL) and were irradiated with the light of a 150 W—high-pressure mercury arc lamp (TQ 150, Heraeus). When the reaction was finished the mixture was concentrated under reduced pressure and purification was accomplished by flash chromatography to give naphthalenophanes **7** and **8**.

*3,4-Diacetyl-12,17-dioxatetracyclo[17.3.1.1^6,10^.0^2,7^]tetracosa-1(23),2,4,6,6(24),7,9,19,21-nonaene-11,18-dione* (**7b**). IR (KBr, cm^−1^): 3017, 2961, 1718, 1606, 1248, 889, 747. ^1^H-NMR (CDCl_3_, ppm): δ 3.59–3.68 (2H, *m*, CH_2_); 3.87–3.94 (2H, *m*, CH_2_); 4.09–4.23 (4H, *m*, CH_2_); 7.39–7.43 (2H, *m*, CH_Ar_); 7.58–7.63 (1H, *m*, CH_Ar_); 7.66–7.70 (1H, *m*, CH_Ar_); 7.88–7.92 (1H, *m*, CH_Ar_); 7.98–8.02 (1H, *m*, CH_Ar_); 8.23 (1H, *s*, CH_Ar_); 8.46 (1H, *s*, CH_Ar_). HRMS (ESI) calcd. for C_26_H_22_O_6_+H^+^: 431.1495 found: 431.1498.

*3,4-Diacetyl-12,19-dioxatetracyclo[19.3.1.1^6,10^.0^2,7^]hexacosa-1(25),2,4,6,6(26),7,9,21,23-nonaene-11,20-dione* (**7c**). mp = 275 °C. IR (KBr, cm^−1^): 2922, 2852, 1720, 1632, 1359, 1257, 802, 752. ^1^H-NMR (CDCl_3_, ppm): δ 1.62–1.83 (4H, *m*, CH_2_), 2.09 (3H, *s*, CH_3_), 2.77 (3H, *s*, CH_3_), 4.14–4.25 (3H, *m*, CH_2_), 4.45–4.54 (1H, *m*, CH_2_), 7.54–7.62 (2H, *dd*, CH_Ar_, *^3^J* = 9.3 Hz), 7.68 (1H, *s*, CH_Ar_), 7.80–7.83(1H, *d*, CH_Ar_, *^3^J* = 9.3 Hz), 8.04–8.08 (2H, *m*, CH_Ar_), 8.49 (1H, *s*, CH_Ar_), 8.59 (*s*, 1H, CH_Ar_). ^13^C-NMR (CDCl_3_, ppm): δ 27.14 (CH_3_), 28.36 (CH_2_), 28.86 (CH_2_), 29.71 (CH_2_), 30.32 (CH_2_), 31.23 (CH_3_), 66.26 (CH_2_), 67.40 (CH_2_), 128.15 (CH_Ar_), 128.54 (CH_Ar_), 129.09 (CH_Ar_), 129.16 (CH_Ar_), 129.90 (C_q/Ar_), 129.99 (C_q/Ar_), 130.25 (CH_Ar_), 131.56 (CH_Ar_), 132.19 (C_q/Ar_), 133.26 (C_q/Ar_), 133.77 (CH_Ar_), 135.16 (C_q/Ar_), 135.68 (C_q/Ar_), 136.52 (CH_Ar_), 137.74 (C_q/Ar_), 139.77 (C_q/Ar_), 166.23 (C_q/COO_), 198.09 (C_q/CO_), 205.03 (C_q/CO_). HRMS (ESI) calcd. for C_28_H_6_O_6_+H^+^: 459.1808, found: 459.1822.

*3,4-Diacetyl-12,15,18,21-tetraoxatetracyclo[21.3.1.1^6,10^.0^2,7^]octacosa-1(27),2,4,6,6(28),7,9,23,25-nonaene-11,22-dione* (**7d**). mp = 240 °C. IR (KBr, cm^−1^): 3427, 2926, 1721, 1288, 1243, 1193, 1110, 1080, 752, 668. ^1^H-NMR (CDCl_3_, ppm): δ 1.88 (3H, *s*, CH_3_); 2.59 (3H, *s*, CH_3_); 3.22–3.30 (1H, *m*, CH_2_); 3.35–3.42 (2H, *m*, CH_2_); 3.53–3.60 (3H, *m*, CH_2_); 3.92–3.97 (1H, *m*, CH_2_); 4.20–4.24 (1H, *m*, CH_2_); 4.17–4.21 (2H, *m*, CH_2_); 4.39–4.45 (1H, *m*, CH_2_); 4.58–4.66 (1H, *m*, CH_2_); 7.58–7.63 (1H, *t*, CH_Ar_, ^3^*J* = 7.6 Hz); 7.71–7.76 (2H, *m*, CH_Ar_); 7.95 (1H, *s*, CH_Ar_); 8.11–8.14 (1H, *m*, CH_Ar_); 8.16–8.19 (1H, *m*, CH_Ar_); 8.52 (1H, *s*, CH_Ar_); 8.75 (1H, *s*, CH_Ar_). ^13^C-NMR (CDCl_3_, ppm): δ 27.2 (CH_3_); 31.4 (CH_3_); 64.7 (CH_2_); 66.4 (CH_2_); 69.0 (CH_2_); 69.3 (CH_2_); 69.7 (CH_3_); 71.4 (CH_3_); 127.7 (CH_Ar_); 128.3 (CH_Ar_); 129.3 (CH_Ar_); 129.5 (CH_Ar_); 129.9 (C_q/Ar_); 131.8 (CH_Ar_); 132.0 (CH_Ar_); 134.2 (CH_Ar_); 134.9 (CH_Ar_); 135.4 (C_q/Ar_); 136.5 (C_q/Ar_). HRMS (ESI) calcd. for C_28_H_26_O_8_+H^+^: 491.1706 found: 491.1733.

*3,4-Diacetyl-14,20-dioxatetracyclo[22.3.1.1^6,10^.0^2,7^]nonacosa-1(28),2,4,6,6(29),7,9,24,26-nonaene-13,21-dione* (7e). IR (KBr, cm^−1^): 3353, 2925, 1724, 1639, 1556, 1459, 1250, 1164, 1078, 956, 755, 620. ^1^H-NMR (CDCl_3_, ppm): δ 0.83–1.22 (9H, *m*, CH_2_); 2.12 (3H, *s*, CH_3_); 2.57–2.66 (2H, *m*, CH_2_); 2.72–2.78 (1H, *m*, CH_2_); 2.74 (3H, *s*, CH_3_); 2.80–2.84 (2H, *m*, CH_2_); 2.99–3.05 (3H, *m*, CH_2_) 3.16–2.20 (2H, *t*, CH_2_, ^3^*J* = 6.3 Hz); 3.78–3.83 (2H, *t*, CH_2_, ^3^*J* = 7.4 Hz); 3.84–4.03 (5H, *m*, CH_2_); 7.05 (1H, *s* CH_Ar_); 7.11–7.14 (1H, *d*, CH_Ar_, ^3^*J* = 7.4 Hz); 7.28–7.30 (1H, *d*, CH_Ar_, ^3^*J* = 7.8 Hz); 7.36–7.42 (3H, *m*, CH_Ar_); 7.82 (1H, *s*, CH_Ar_); 8.33 (1H, *s*, CH_Ar_). ^13^C-NMR (CDCl_3_, ppm): δ 21.8 (CH_2_); 27.0 (CH_3_); 27.7 (CH_2_); 28.2 (CH_2_); 30.7 (CH_2_); 30.9 (CH_2_); 31.9 (CH_3_); 35.1 (CH_2_); 35.3 (CH_2_); 126. 7 (CH_Ar_); 128.2 (CH_Ar_); 128.5 (CH_Ar_); 128.6 (CH_Ar_); 129.3 (CH_Ar_); 130.3 (CH_Ar_); 130.5 (CH_Ar_); 130.7 (CH_Ar_); 132.1 (C_q/Ar_); 133.11 (C_q/Ar_); 139.4 (C_q/Ar_); 140.2 (C_q/Ar_); 172.1 (C_q/COO_); 173.2 (C_q/COO_). HRMS (ESI) calcd. for C_31_H_32_O_6_+2H: 502.2355 found: 502.2332.

*21,22-Diacetyl-8,13-dioxatetracyclo[13.7.1.1^2,6^.0^19,23^]tetracosa 1(23),2(24),3,5,15(23),16,18,19,21-nonaene-7,14-dione* (**8b**) mp 148–151 °C. IR (KBr, cm^−1^): 3017, 2961, 1718, 1606, 1248, 889, 747. ^1^H-NMR (CDCl_3_, ppm): δ 1.73–1.79 (2H, *m*, CH_2_); 1.97 (3H, *s*, CH_3_); 2.17–2.26 (2H, *m*, CH_2_); 2.76 (3H, *s*, CH_3_); 3.30–3.37 (1H, *t*, CH_2_, *^2^J* = 10.5 Hz); 3.95–4.00 (1H, *m*, CH_2_); 4.21–4.25 (1H, *m*, CH_2_); 4.75–4.82 (1H, *t*, CH_2_, *^2^J* = 10.4 Hz); 7.56–7.58 (2H, *m*, CH_Ar_); 7.64–7.67 (1H, *d*, CH_Ar_, *^3^J* = 8.0 Hz); 7.77–7.80 (1H, *dd*, CH_Ar_, *^3^J* = 7.2 Hz, *^4^J* = 1.4 Hz); 8.04 (1H, *s*, CH_Ar_); 8.05–8.09 (1H, *m*, CH_Ar_); 8.12–8.15 (1H, *dd*, CH_Ar_, *^3^J* = 8.1 Hz, ^4^*J* = 1.3 Hz); 1.40 (1H, *s*, CH_Ar_). ^13^C-NMR (CDCl_3_, ppm): δ 24.0 (CH_2_); 26.7 (CH_2_); 27.5 (CH_3_); 31.7 (CH_3_); 64.3 (CH_2_); 65.8 (CH_2_); 126. 2 (CH_Ar_); 129.3 (CH_Ar_); 129.4 (CH_Ar_); 130.4 (CH_Ar_) 130.6 (C_q/Ar_); 131.6 (CH_Ar_); 132.3 (C_q/Ar_); 132.4 (CH_Ar_); 132.8 (CH_Ar_); 133.5 (C_q/Ar_);136.2 (CH_Ar_); 137.7 (C_q/Ar_); 141.8 (C_q/COO_); 155.7 (C_q/COO_); 166.6 (C_q/CO_); 168.1 (C_q/CO_). HRMS (ESI) calcd. for C_26_H_22_O_6_+H^+^: 431.1495 found: 431.1511.

*23,24-Diacetyl-8,15-dioxatetracyclo[15.7.1.1^2,6^.0^21,25^]hexacosa-1(25),2(26),3,5,17(25),18,20,21,23-nonaene-7,16-dione* (**8c**) mp 155–158 °C. IR (KBr, cm^−1^): 3018, 2931, 2857, 1716, 1658, 1352, 1255, 889, 751. ^1^H-NMR (CDCl_3_, ppm): δ 1.22–1.27 (1H, *m*, CH_2_); 1.45–1.61 (5H, *m*, CH_2_); 1.74–1.86 (2H, *m*, CH_2_); 1.93 (3H, *s*, CH_3_); 2.77 (1H, *s*, CH_3_); 3.01-3.05 (1H, *m*, CH_2_); 3.76–3.80 (1H, *m*, CH_2_); 4.33–39 (1H, *m*, CH_2_); 4.59–4.67 (1H, *m*, CH_2_); 7.52–7.54 (2H, *m*, CH_Ar_); 7.62–7.67 (1H, *m*, CH_Ar_); 7.73–7.76 (1H, *dd*, CH_Ar_, *^3^J* = 7.1 Hz, *^4^J* = 1.4 Hz); 7.94 (1H, *s*, CH_Ar_); 8.10–8.15 (2H, *m*, CH_Ar_); 8.46 (1H, *s*, CH_Ar_). ^13^C-NMR (CDCl_3_, ppm): δ 23.8 (CH_2_); 24.7 (CH_2_); 25.2 (CH_2_); 25.8 (CH_2_); 27.24 (CH_3_); 31.5 (CH_3_); 65.0 (CH_2_); 66.0 (CH_2_); 126.4 (CH_Ar_); 128.5 (CH_Ar_); 129.5 (CH_Ar_); 130 (C_q/Ar_); 131.4 (CH_Ar_); 131.5 (CH_Ar_); 131.7 (CH_Ar_); 131.9 (C_q/Ar_); 132.2 (CH_Ar_); 133.1 (C_q/Ar_); 136.7 (CH_Ar_); 137.1 (C_q/Ar_). HRMS [ESI] calcd. for C_28_H_26_O_6_+H^+^: 459.1808 found: 459.1775.

*25,26-Diacetyl-8,11,14,17-tetraoxatetracyclo[17.7.1.1^2,6^.0^23,27^]octacosa-1(27),2(28),3,5,19(27),20,22,23,25-nonaene-7,18-dione* (**8d**) mp 182–184 °C. IR (KBr, cm^−1^): 3369, 2925, 1717, 1359, 1253, 1115, 930, 750, 665. ^1^H-NMR (CDCl_3_, ppm): δ 1.90 (3H, *s*, CH_3_); 2.74 (3H, *s*, CH_3_); 3.19–3.27 (1H, *m*, CH_2_); 3.58–3.84 (9H, *m*, CH_2_); 4.37–4.40 (1H, *m*, CH_2_); 4.72–4.80 (1H, *m*, CH_2_); 7.47–7.72 (4H, *m*, CH_Ar_); 7.95 (1H, *s*, CH_Ar_); 8.08–8.13 (2H, *m*, CH_Ar_); 8.43 (1H, *s*, CH_Ar_). ^13^C-NMR (CDCl_3_, ppm): δ 27.2 (CH_3_); 31.5 (CH_3_); 63.0 (CH_2_); 65.0 (CH_2_); 68.7 (CH_2_); 68.9 (CH_2_); 70.4 (CH_2_); 71.5 (CH_2_); 126.4 (CH_Ar_); 128.3 (CH_Ar_); 129.6 (CH_Ar_); 131.5 (CH_Ar_); 131.7 (CH_Ar_); 132.0 (CH_Ar_); 132.2 (CH_Ar_); 133.0 (C_q/Ar_); 136.7 (CH_Ar_); 136.9 (C_q/Ar_). HRMS (ESI) calcd. for C_28_H_26_O_8_+H^+^: 491.1706 exp.: 491.1720.

*26,27-Diacetyl-10,16-dioxatetracyclo[18.7.1.1^2,6^.0^24,28^]nonacosa-1(28),2(29),3,5,20(28),21,23,24,26-nonaene-9,17-dione* (**8e**) IR (KBr, cm^−1^): 2926, 1727, 1681, 1350, 1250, 1448, 970, 832, 754, 710, 667. ^1^H-NMR (CDCl_3_, ppm): δ 1.50–1.65 (6H, *m*, CH_2_), 2.12 (3H, *s*, CH_3_); 2.58–2.66 (2H, *m*, CH_2_); 2.74 (3H, *s*, CH_3_); 2.77–2.84 (2H, *m*, CH_2_); 2.99–3.05 (2H, *m*, CH_2_); 3.16–3.20 (2H, *t*, CH_2_, *^3^J* = 6.3 Hz); 3.78–3.83 (2H, *t*, CH_2_, *^3^J* = 7.4 Hz); 3.95–4.06 (4H, *m*, CH_2_); 7.00–7.05 (1H, *m*, CH_Ar_); 7.10–7.11 (1H, *d*, CH_Ar_, *^3^J* = 7.4 Hz); 7.28–7.30 (2H, *m*, CH_Ar_); 7.36–7.39 (2H, *m*, CH_Ar_); 7.83 (1H, *s*, CH_Ar_); 8.33 (1H, *s*, CH_Ar_). ^13^C-NMR (CDCl_3_, ppm): δ 22.2 (CH_2_); 23.3 (CH_2_); 27.4 (CH_3_); 28.1 (CH_2_); 28.6 (CH_2_); 31.1 (CH_2_); 31.3 (CH_2_); 32.5 (CH_3_); 35.67 (CH_2_); 63.9 (CH_2_); 64.8 (CH_2_); 127.1 (CH_Ar_); 128.6 (CH_Ar_); 128.9 (CH_Ar_); 129.0 (CH_Ar_); 129.7 (CH_Ar_); 130.9 (CH_Ar_); 131.2 (CH_Ar_); 131.6 (CH_Ar_); 132.5 (C_q/Ar_); 138.3 (C_q/Ar_); 139.8 (C_q/Ar_); 140.0 (C_q/Ar_); 140.6 (C_q/Ar_); 141.3 (C_q/Ar_). HRMS (ESI) calcd. for C_31_H_32_O_6_+Na^+^: 523.2097 found: 523.2110.

*3,4-Diacetyl-14,17,20,23-tetraoxatetracyclo[25.3.1.1^6,10^.0^2,7^]dotriaconta-1(31),2,4,6,6(32),7,9,27,29-nonaene-13,24-dione* (**7f**) and *29,30-Diacetyl-10,13,16,19-tetraoxatetracyclo [21.7.1.1^2,6^.0^27,31^]-dotriaconta-1(31),2(32),3,5,23(31),24,26,27,29-nonaene-9,20-dione* (**8f**). HRMS (ESI) calcd for C_32_H_34_O_8_+H^+^: 547.2332 found: 547.2336.

## 4. Conclusions

In summary, we have reported on the Photo-Dehydro-Diels-Alder (PDDA) reaction of various diketones **6** to give mixtures of 1,6- and 1,8-naphthalenophanes **7** and **8** with yields depending on the length of the chain bridging the naphthalene moiety. The PDDA reactants could be efficiently prepared in three steps starting with ω-(3-iodophenyl)-carboxylic acids **1**. The product mixtures could be separated in almost all cases by flash chromatography. Because the biaryl moiety of naphthalenophanes possess a chirality axis the question of configurational stability at room temperature arose in which the height of the rotational barrier influences this phenomenon. For the determination of this barrier two methods are appropriate: dynamic HPLC (DHPLC) and dynamic NMR (DNMR) experiments. Whereas naphthalenophanes with the shortest chain (compounds **7b**, **8b**) are configurationally stable, the products with a longer chain undergo atropisomerization depending on the temperature. The determined rotational barriers range from 19.6 kcal/mol (**7d**) to 22.5 kcal/mol (**8e**).

## Figures and Tables

**Table 1:** Synthesis of bisynones **6**.

Product	n	HO-R-OH	Yield 3 [%]	Yield 5 [%]	Yield 6 [%]
6a	0	HO-(CH_2_)_2_-OH	97	97	90
6b	0	HO-(CH_2_)_4_-OH	69	95	86
6c	0	HO-(CH_2_)_6_-OH	93	92	85
6d	0	HO-(CH_2_CH_2_O)_3_-H	99	93	62
6e	2	HO-(CH_2_)_5_-OH	100	100	100
6f	2	HO-(CH_2_CH_2_O)_3_-H	90	93	67

**Table 2:** Yields of naphthalenophanes **7** and **8**.

Reactant	n	k ^(a)^	Yield 7 [%]	Yield 8 [%]	Yield 7 + 8 [%]
6a	0	9	0	0	0
6b	0	11	3	12	15
6c	0	13	15	26	41
6d	0	15	33	33	66
6e	2	16	^(b)^	^(b)^	21
6f	2	19	^(b)^	^(b)^	61

(a) overall number of atoms forming the chain of naphthalenophanes; (b) individual yields could not be determined due to insufficient separation of products and superposition of signals in the NMR spectra.

**Table 3:** Barrier of atropisomerization of **7** and **8**.

Compound	DHPLC *ΔG_ROT_* [kcal/mol]	DNMR *ΔG_ROT_* [kcal/mol]
7b	-	-
7c	-	20.6
7d	-	19.6
7e	20.4	-
8b	-	-
8c	<20.0	≈20.0
8d	21.5	-
8e	22.5	-

## References

[1] Gleiter R., Hopf H. (2004). Modern Cyclophane Chemistry.

[2] Bogdan N.D., Grosu I. (2009). [4.n]cyclophanes. Curr. Org. Chem..

[3] Vögtle F., Schäfer R., Schunder L., Neumann P. (1970). Steric interactions of inner atoms in cyclic compounds. XIII. Naphthalenophanes. Liebigs Ann. Chem..

[4] Vögtle F., Neumann P. (1969). Nomenclature of phanes [cyclic bridged aromatic compounds]. Tetrahedron Lett..

[5] Wessig P., Matthes A. (2011). Preparation of Strained Axially Chiral (1,5)Naphthalenophanes by Photo-dehydro-Diels-Alder Reaction. J. Am. Chem. Soc..

[6] Wessig P., Müller G., Pick C., Matthes A. (2007). The Photo-Dehydro-Diels–Alder Reaction for the Preparation of Biaryls. Synthesis.

[7] Wessig P., Matthes A., Pick C. (2011). The Photo-dehydro-Diels-Alder (PDDA) reaction. Org. Biomol. Chem..

[8] Wessig P., Müller G. (2008). The Dehydro-Diels-Alder Reaction. Chem. Rev..

[9] Wessig P., Müller G., Kühn A., Herre R., Blumenthal H., Troelenberg S. (2005). The Photo-Dehydro-Diels-Alder Reaction: An efficient route to naphthalenes. Synthesis.

[10] Wessig P., Müller G. (2006). Synthesis of 1,1'-Binaphthyls by Photo-Dehydro-Diels-Alder reaction. Chem. Commun..

[11] Wessig P., Müller G., Herre R., Kühn A. (2006). Synthesis of Benzo[g]isochromenes through Photo-Dehydro-Diels-Alder Reaction. Helv. Chim. Acta.

[12] Wessig P., Müller G. (2008). Facile Photochemical Synthesis of 1,1'-Binaphthyls. Aust. J. Chem..

[13] Wessig P., Pick C. (2011). Photochemical synthesis and properties of axially chiral naphthylpyridines. J. Photochem. Photobiol. A.

[14] Kawasaki N., Goto M., Kawabata S., Kometani T. (2001). The effect of vinyl esters on the enantioselectivity of the lipase-catalysed transesterification of alcohols. Tetrahedron: Asymmetry.

[15] Neises B., Steglich W. (1978). Simple Method for the Esterification of Carboxylic Acids. Angew. Chem. Int. Ed..

[16] Sonogashira K., Tohda Y., Hagihara N. (1975). A convenient synthesis of acetylenes: Catalytic substitutions of acetylenic hydrogen with bromoalkenes, iodoarenes and bromopyridines. Tetrahedron Lett..

[17] Dess D., Martin B. (1983). Readily accessible 12-I-5 oxidant for the conversion of primary and secondary alcohols to aldehydes and ketones. J. Org. Chem..

[18] Dess D., Martin B. (1991). A useful 12-I-5 triacetoxyperiodinane (the Dess-Martin periodinane) for the selective oxidation of primary or secondary alcohols and a variety of related 12-I-5 species. J. Am. Chem. Soc..

[19] Cope A.C., Martin M.M., McKervey M.A. (1966). Transannular reactions in medium-sized rings. Q. Rev. Chem. Soc..

[20] Bringmann G., Gulder T., Gulder T.A.M., Breuning M. (2011). Atroposelective total synthesis of axially chiral biaryl natural products. Chem. Rev..

[21] Bringmann G., Mortimer A.J.P., Keller P.A., Gresser M.J., Garner J., Breuning M. (2005). Atroposelective synthesis of axially chiral biaryl compounds. Angew. Chem. Int. Edit..

[22] Sandström J. (1982). Dynamic NMR Spectroscopy.

[23] Trapp O. (2006). Fast and precise access to enantiomerization rate constants in dynamic chromatography Chirality. Chirality.

